# Enhanced the Overall Water Splitting Performance of Quaternary NiFeCrCo LDH: Via Increasing Entropy

**DOI:** 10.3390/molecules30071461

**Published:** 2025-03-25

**Authors:** Xin Liu, Li Bai, Xinrong Guo, Haoyu Li, Xiaoyan Liu, Jian Cao, Lili Yang, Maobin Wei, Yanli Chen, Huilian Liu, Qiang Tao

**Affiliations:** 1 Key Laboratory of Functional Materials Physics and Chemistry of the Ministry of Education, Jilin Normal University, Changchun 130103, China; 15330783861@163.com (X.L.); 15326575020@163.com (L.B.); 17335887075@163.com (X.G.); 15568559566@163.com (H.L.); liuxiaoyan1437@163.com (X.L.); caojian_928@163.com (J.C.); llyang1980@126.com (L.Y.); jlsdzccw@126.com (M.W.); 2 National Demonstration Center for Experimental Physics Education, Jilin Normal University, Siping 136000, China; 3 Synergetic Extreme Condition High-Pressure Science Center, State Key Laboratory of Superhard Materials, College of Physics, Jilin University, Changchun 130012, China; qiangtao@jlu.edu.cn

**Keywords:** NiFeCrCo LDH, one-step hydrothermal method, entropy, electrocatalyst, overall water splitting

## Abstract

The construction of high-performance catalysts for overall water splitting (OWS) is crucial. Nickel–iron-layered double hydroxide (NiFe LDH) is a promising catalyst for OWS. However, the slow kinetics of the HER under alkaline conditions seriously hinder the application of NiFe LDH in OWS. This work presents a strategy to optimize OWS performance by adjusting the entropy of multi-metallic LDH. Quaternary NiFeCrCo LDH was constructed, which exhibited remarkable OWS activity. The OER and HER of NiFeCrCo LDH were stable for 100 h and 80 h, respectively. The OWS activity of NiFeCrCo LDH//NiFeCrCo LDH only required 1.42 V to reach 10 mA cm^−2^, and 100 mA cm^−2^ required 1.54 V. Under simulated seawater conditions, NiFeCrCo LDH//NiFeCrCo LDH required 1.57 V to reach 10 mA cm^−2^ and 1.71 V to reach 100 mA cm^−2^. The introduction of Co into the structure induced Cr to provide more electrons to Fe, which regulated the electronic state of NiFeCrCo LDH. The appropriate electronic state of the structure is essential for the remarkable performance of OWS. This work proposes a new strategy to achieve excellent OWS performance through entropy-increase engineering.

## 1. Introduction

Electrocatalytic water splitting is an effective solution for environmental pollution and energy shortages [1,2]. This process involves two half-reactions: the hydrogen evolution reaction (HER) at the cathode and oxygen evolution reaction (OER) at the anode. Suitable catalysts can significantly reduce the energy barrier associated with these reactions and enhance the reaction efficiency [3,4,5]. Although precious metals such as platinum (Pt), ruthenium (Ru), and iridium (Ir) exhibit high catalytic activity, their limited availability and high cost restrict their potential for large-scale applications [6,7]. Therefore, it is more meaningful to develop alternative, abundant catalysts that can provide comparable performance to that of precious metal catalysts.

Among many non-precious metal electrocatalysts, nickel–iron-layered double hydroxide (NiFe LDH) has shown considerable potential because of its abundant reserves, two-dimensional layered structure, and adjustable interlayer anions [8,9,10,11,12,13,14]. For instance, Liu et al. simulated the introduction of different anion intercalations between NiFe LDH layers through theoretical calculations, and the results showed that interlayer anions can regulate catalytic activity and promote OER activity through essential charge transfer [15]. Jong Hyeok Park’s team used the large specific surface area of NiFe LDH itself to grow Co_3_O_4_, thereby promoting the oxidation of the Ni^2+^ state on the inert surface of NiFe LDH to the more catalytically active Ni^3+^ state, enhancing OER performance and demonstrating a high current density of 100 mA cm^−2^ at 1.48 V [16]. However, three important obstacles need to be addressed. Firstly, the layered structure of NiFe LDH results in poor electrical conductivity, hindering the transfer of electrons and reducing the reaction rate. Secondly, NiFe LDH is prone to aggregation and structural degradation during long-term operation, reducing its stability. Thirdly, the slow kinetics of the HER under alkaline conditions seriously hinder its application in overall water splitting (OWS) [17,18].

Various optimization strategies have been devised to improve the overall catalytic activity of NiFe LDH, such as heteroatom doping, defect engineering, and interface engineering [19,20,21]. With these approaches, the catalytic activity of the catalysts can be effectively improved through entropy-increase engineering. It is well known that an increase in the entropy value can induce unique properties that make them attractive for catalytic application. Firstly, the high entropy effect and sluggish diffusion effect can enhance the stability of the material itself and improve the durability of the catalyst during the catalytic reaction [22,23,24]. Secondly, lattice distortion can introduce microstructures/defects into the catalyst, reconstitute the electronic structure, and increase the density of the active sites of the catalyst [25]. Thirdly, the cocktail effect can yield unique electronic and catalytic properties by utilizing the synergistic effect between the various metal elements in the structure [26,27]. For example, the OER activity of high-entropy CoFeMnCuZn (oxy)hydroxide is better than that of FeCo and FeCoZn hydroxides; it can induce the O 2p band center to move towards the Fermi level through the presence of Zn in high-entropy CoFeMnCuZn (oxy)hydroxide, resulting in more Co^4+^ species in the structure, which drives holes in the oxygen ligands and promotes intramolecular oxygen coupling [28]. A report shows that the adjustment of entropy in catalysis can be used to optimize the catalytic activity, in which the material structure is modified from various aspects, and performance can be improved. Therefore, by combining entropy-increase engineering with NiFe LDH, the advantages of both can be combined to create satisfactory catalysts. These catalysts may be beneficial for industrial applications in the future due to their high performance (high conductivity, activity of active sites), low cost, and stability for long-term service.

In this study, a novel strategy for entropy engineering using NiFe LDH is proposed, and the obstacles of poor conductivity, low stability, and slow kinetics of the HER in NiFe LDH are addressed. A facile one-step hydrothermal method is proposed for the synthesis of multi-metallic LDH based on the NiFe LDH structure. A systematic synthesis is carried out by introducing different metal ions with +2 and/or +3 valence states (such as Co, Cu, Zn, Mn, Cr, Al, and V) into NiFe LDH to obtain different ternary and quaternary LDHs. The introduction of different valence metal ions can affect the catalytic performance of the materials. The results show that quaternary NiFeCrCo LDH exhibits excellent HER, OER, and OWS performance in alkaline conditions and has good catalytic activity in simulated seawater. Finally, the synergistic effect between metal elements increases the conductivity, stability, and activity of the active sites of LDH. This investigation paves the way for the design of LDHs for efficient and stable electrocatalysis.

## 2. Results and Discussion

### 2.1. Subsection Electrocatalysts Characterization

**Scheme 1:**
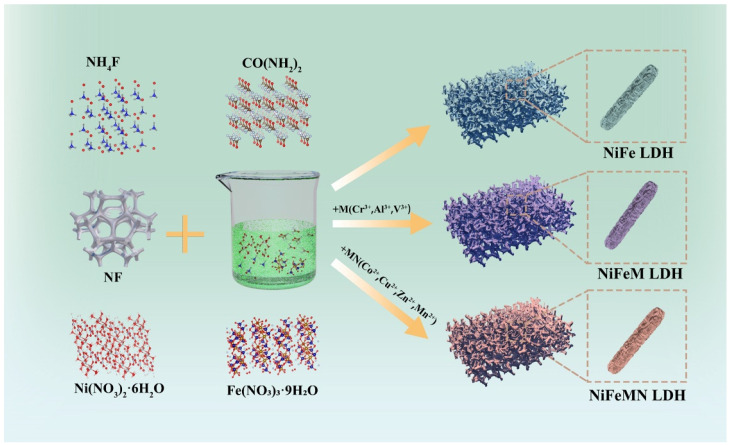
Schematic illustration of the synthesis of ternary and quaternary LDH.

A facile one-step hydrothermal method for synthesizing different ternary and quaternary LDH electrocatalysts as an advanced OWS electrocatalyst was proposed, as illustrated in Scheme 1. Ternary or quaternary LDH can be obtained by introducing different +3 valent state or +2 valent state metal ions into the process of NiFe LDH synthesis. Since metal ions of the same valence state in the structure have equal stoichiometric ratios, the introduction of such metal ions is not doping but is similar to a solid solution.

**Figure 1:**
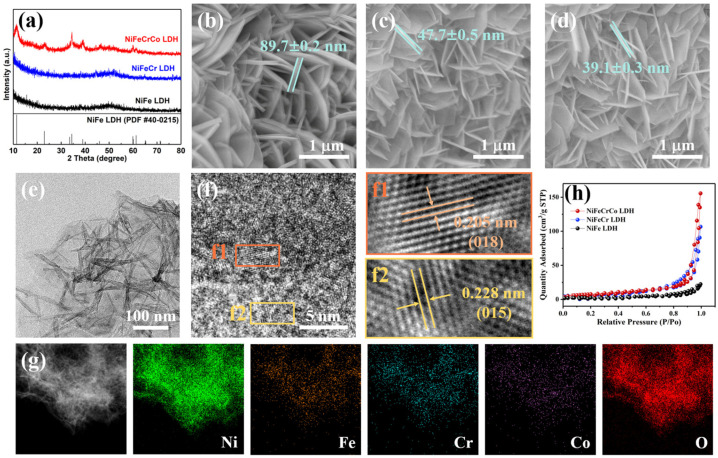
(**a**) XRD patterns of quaternary NiFeCrCo LDH, (**b**) SEM image of NiFe LDH, (**c**) SEM image of ternary NiFeCr LDH, (**d**) SEM image of quaternary NiFeCrCo LDH, (**e**) TEM image of quaternary NiFeCrCo LDH, (**f**) HRTEM image of quaternary NiFeCrCo LDH, (**f1**) and (**f2**) are the zoomed-in images taken from the frame orange area and frame yellow area marked in (**f**), (**g**) HAADF-STEM image of quaternary NiFeCrCo LDH, followed by the correponding elemental maps, (**h**) N_2_ adsorption/desorption isotherms of NiFe LDH, NiFeCr LDH and NiFeCrCo LDH.

X-ray diffraction (XRD) characterization of the quaternary NiFeCrCo LDH was carried out to determine its crystal structure. The NiFeCrCo LDH/Nickel Foam (NF) was first probed, as shown in Figure S1. Three strong diffraction peaks are observable at 44.5°, 51.9°, and 76.4°, which are characteristic of metal Ni (JCPDS No. 4–0850). This is possibly because the diffraction peak of metal Ni is too strong, resulting in the diffraction peak of NiFeCrCo LDH being submerged [29,30]. Figure 1a shows the XRD patterns of the powders stripped from the NF substrate. NiFe LDH has poor crystallinity (7.5%), and the crystallinities of NiFeCr LDH and NiFeCo LDH are 58.5% and 60.6%, respectively. Introducing Cr and Co can improve the crystallinity. The diffraction peaks of NiFeCrCo LDH at 11.4°, 22.9°, 34.4°, 39.0°, 45.9°, 59.6°, and 61.3° correspond to the (003), (006), (012), (015), (018), (110), and (113) crystal planes, respectively (JCPDS No. 40–0215) [31,32]. The diffraction peaks do not shift after the addition of Cr and Co, because the atomic radii of Cr and Co are about the same as the atomic radii of Fe and Ni; therefore, the addition of Cr and Co does not cause the expansion or contraction of the lattice in the structure.

The SEM images of NiFe LDH, NiFeCr LDH, and NiFeCrCo LDH are shown in Figure 1b–d. The morphologies of the three LDHs are ultra-thin nanosheets growing vertically on the NF substrate. Observations show that the thickness of the nanosheets gradually becomes smaller as the variety of metal elements in the structure increases. This thinning can also be observed in the TEM images (Figure 1e, Figures S2a, S3a and S4a), which results in curling at the edges of the nanosheets of NiFeCr LDH (Figure S3a) and NiFeCrCo LDH (Figure S4a). The microstructures of NiFe LDH, NiFeCr LDH, and NiFeCrCo LDH were analyzed using HRTEM. As can be seen from Figure 1(f1,f2) and Figure S4, there is a regular lattice arrangement in NiFeCrCo LDH, and the lattice fringes of 0.227 and 0.205 nm correspond to the (015) and (018) crystallographic planes. The lattice distances are in agreement with the XRD results (Figure 1a). In addition, the distribution of elements in the NiFeCrCo LDH was analyzed using high-angle annular dark-field scanning transmission electron microscopy (HAADF-STEM) and elemental mapping. The results are shown in Figure 1g. The images demonstrate the uniform distribution of Ni, Fe, Cr, Co, and O elements throughout the nanosheets. The same characterizations were performed for NiFe LDH and NiFeCr LDH, and the results are shown in Figures S2 and S3. Regular lattice fringes were observed in NiFe LDH and NiFeCr LDH, and the various elements were evenly distributed in the nanosheets. This indicates that the one-step hydrothermal method can successfully synthesize multicomponent LDHs.

The specific surface areas of the three LDHs were determined using N_2_ adsorption/desorption isotherms. It can be observed from Figure 1h that all of the samples belong to the type-IV shape with a hysteresis loop, indicating the presence of a porous structure in the samples. The calculation results show that the BET specific surface area of NiFeCrCo LDH is 28.6 m^2^·g^−1^, which is larger than that of NiFe LDH (8.7 m^2^·g^−1^) and NiFeCr LDH (23.6 m^2^·g^−1^). The introduction of the third element and/or the fourth element can inhibit the growth of the grain and form a thinner nanosheet structure; thus, the quaternary LDH displays a larger specific surface area, and the results are consistent with the SEM and TEM results (Figure 1b–e and Figures S2–S4).

**Figure 2:**
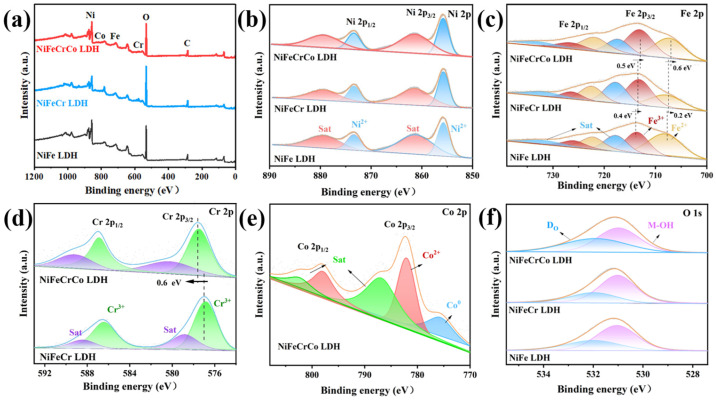
(**a**) XPS survey spectra of NiFe LDH, NiFeCr LDH, and NiFeCrCo LDH, high-resolution XPS spectra of (**b**) Ni 2p, (**c**) Fe 2p, (**d**) Cr 2p, (**e**) Co 2p, and (**f**) O 1s.

The surface chemical states and charge transfer of the samples were investigated using X-ray photoelectron spectroscopy (XPS). The XPS survey spectra of NiFe LDH, NiFeCr LDH, and NiFeCrCo LDH are shown in Figure 2a, in which the presence of Ni, Fe, Cr, Co, and O in NiFeCrCo LDH is evident. The high-resolution Ni 2p spectra show that the peaks at 873.4 and 855.8 eV belong to Ni^2+^, and the peaks at 879.3 and 861.2 eV belong to the Ni 2p satellite peaks (Figure 2b) [33]. The peaks of Ni 2p in the three samples did not change, indicating that the introduction of Cr and Co did not cause charge transfer to Ni. In the NiFe LDH, the peaks at 722.2 and 708.1 eV belong to Fe^2+^, and 725.9 and 713.6 eV belong to Fe^3+^, along with two satellite peaks at 731.8 and 717.7 eV (Figure 2c). The peaks of Fe 2p shifted significantly after the introduction of Cr and Co. After the introduction of Cr, the peaks of Fe^2+^ and Fe^3+^ shifted 0.2 and 0.4 eV to the low binding energy position, indicating that Cr transferred electrons to Fe. In the NiFeCrCo LDH, where Co was introduced, the peaks of Fe^2+^ and Fe^3+^ continued to move towards lower binding energies, indicating that Fe could continue to obtain electrons from its surroundings. By analyzing the Cr 2p peaks of NiFeCr LDH and NiFeCrCo LDH, Cr^3+^ at positions 586.4 and 576.9 eV can be observed (Figure 2d) [34,35]. After the introduction of Co, the Cr 2p peaks shifted 0.6 eV towards the high binding energy, indicating that Cr can provide electrons to Co. The charge transfer between metals can be explained by electronegativity: Cr is the least electronegative, and when introduced, it easily provides electrons to Fe. When the more electronegative Co is introduced, Cr continues to transfer electrons outward to Fe and Co. This results in Fe moving towards low-binding energy and Cr moving towards high-binding energy, causing the reconstruction of the electronic structure in the LDH. The peaks of Co 2p are shown in Figure 2e: the peak at 775.6 eV belongs to Co^0^ [36,37], the peaks at 797.9 and 782.1 eV belong to Co^2+^, and two satellite peaks are observable at 802.3 and 786.8 eV [38,39]. The ability of Cr to donate electrons is limited when more Co is introduced. As a result, some parts of Co can only exist in the Co⁰ state at specific local positions. The O 1s spectra are shown in Figure 2f, where the peak at 531.0 eV is assigned to the hydroxide groups (M–OH), and the peak at 531.9 is assigned to the oxygen defects on the surface (D_o_) [40]. With an increase in the number of metal elements in the structure, the content of D_o_ increases, which means that more catalytic active sites may be generated on the surface of the LDH, making it conducive to the catalytic reaction for OWS.

In this work, NiFe LDH has a Ni:Fe ratio of 2:1 (in moles), and the details are described in the “Materials and Methods”. Ni atoms and Fe atoms randomly occupy the metal positions in the layers. When Cr is introduced to form NiFeCr LDH, since both Cr and Fe have an oxidation state of +3, Cr atoms randomly substitute Fe atoms with an equal mole fraction to form a Fe:Cr ratio of 1:1. Therefore, the configurational entropy (*S_conf_*) for the positions of Fe increases to its maximum, and the overall *S_conf_* of NiFeCr LDH increases. Then, since both Co and Ni are +2, Co substitutes Ni atoms with an equal mole fraction to form a Ni:Co ratio of 1:1 in NiFeCrCo LDH. Consequently, the *S_conf_* for the positions of Ni increases to its maximum, and the overall *S_conf_* of NiFeCrCo LDH increases further. Thus, we can infer that the *S_conf_* increases in the order of NiFe LDH, NiFeCr LDH, and NiFeCrCo LDH (Figure S5). Moreover, because these materials are in nano size, the *S_conf_* is also influenced by the surface effect, which should be the configurational entropy of nanomaterials (*S_conf(n)_*). We estimate the order of *S_conf(n)_* of NiFe LDH, NiFeCr LDH, and NiFeCrCo LDH by considering the surface effect in the Supporting Information, which also indicates that the *S_conf(n)_* increases in the order of NiFe LDH, NiFeCr LDH, and NiFeCrCo LDH.

### 2.2. HER and OER Performance

All electrochemical measurements were performed using a standard three-electrode system in a 1 M KOH solution. Different ternary LDHs were formed by mixing different divalent or trivalent metal ions with NiFe LDH, such as NiFeZn LDH, NiFeMn LDH, and NiFeCr LDH. The linear sweep voltammetry (LSV) curves for the HER are shown in Figure S6a,b. The results indicate that the HER activity is improved after the NiFe LDH is mixed with divalent or trivalent metal ions. Among all the ternary LDHs, NiFeCr LDH exhibits the most excellent HER activity, with an overpotential of 216 mV at a current density of 10 mA cm^−2^. Meanwhile, the OER activity of NiFeCr LDH is also very significant, and only requires 357 mV to achieve a current density of 100 mA cm^−2^ (Figure S6c,d). Quaternary LDH can be obtained by mixing metal ions of different valence states in NiFeCr LDH, such as NiFeCrZn LDH, NiFeCrMn LDH, and NiFeCrCo LDH. It can be observed from Figure S7 that the performance of the quaternary LDH is better than that of the ternary LDH, and the introduction of divalent Co ions can further enhance the activity of the catalyst for the OER and HER.

**Figure 3:**
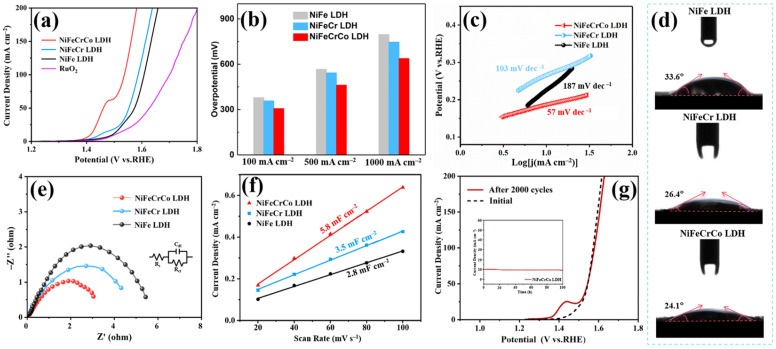
(**a**) Polarization curves of NiFe LDH, NiFeCr LDH, NiFeCrCo LDH, and RuO_2_ in 1 M KOH electrolyte for OER, (**b**) comparison overpotential of NiFe LDH, NiFeCr LDH, and NiFeCrCo LDH at 100, 500 and 1000 mA cm^−2^ in 1 M KOH, (**c**) tafel slope plots, (**d**) the water contact angle results of NiFe LDH, NiFeCr LDH, and NiFeCrCo LDH, (**e**) nyquist plots, (**f**) the double-layer capacitances, and (**g**) the polarization curves of NiFeCrCo LDH before and after 2000 cycles. The insert shows the I-t curve of the OER at 10 mA cm^−2^ for NiFeCrCo LDH.

The more types of metal elements in the structure of LDH, the better the catalytic performance. At a current density of 100 mA cm^−2^, NiFeCrCo LDH has the lowest overpotential (307 mV), which is lower than that of NiFe LDH (379 mV) and NiFeCr LDH (357 mV) (Figure 3a,b). NiFeCrCo LDH also exhibits satisfactory OER performance under high current density conditions (Figure 3b and Figure S8). The HER properties were also compared, as shown in Figure S9. Under both low- and high-current density conditions, NiFeCrCo LDH is still the catalyst with the best performance. The corresponding Tafel slopes indicate that NiFe LDH, NiFeCr LDH, and NiFeCrCo LDH are 187, 103, and 57 mV dec^−1^, respectively (Figure 3c). This indicates that NiFeCrCo LDH has the fastest electron transfer rate [41,42]. NiFeCrCo LDH has the smallest contact angle and the best hydrophilicity (Figure 3d). In the catalytic reaction process, it can make good contact with the electrolyte and promote more active sites to participate in the reaction. The high wetting ability of the electrocatalyst surface also facilitates the rapid separation of bubbles from the electrode surface, and the ultra-large pores (200–500 µm) in the NF substrate also allow for the rapid mass transport and dissipation of gaseous bubbles [43,44,45,46]. In order to investigate the intrinsic OER activity of each active site, we further calculated the O_2_ turnover frequency (TOF) of different LDHs at different overpotentials (Figure S10a). As shown in Figure S10b, when the overpotential is 300 mV vs. RHE, the TOF value of NiFeCrCo LDH is 11.2 s^−1^, while the TOF values of NiFe LDH and NiFeCr LDH are only 6.4 and 7.9 s^−1^, respectively. The results show that NiFeCrCo LDH exhibits the best intrinsic activity.

Electrochemical impedance spectroscopy is used to study the electron transport capacity in catalytic reactions. The semicircle in the high-frequency region indicates the charge transfer resistance (R_ct_), and the smaller the semicircle, the faster the charge transfer in the catalyst. The Nyquist plots of the different LDHs are presented in Figure 3e. NiFeCrCo LDH has the smallest semicircle with an R_ct_ of 3.1 Ω. This indicates that NiFeCrCo LDH has the strongest charge transfer ability during the reaction. The conductivity of NiFe LDH is dominated by the electron-transfer ability within the layers because the interlayer coupling is weak in NiFe LDH. According to the XPS results (Figure 2), the Cr atoms in NiFeCr LDH can offer more electrons to the Fe atoms, and the Co atoms in NiFeCrCo LDH can induce the Cr atoms to offer even more electrons to the Fe atoms. This indicates that introducing Cr and Co into NiFe LDH can generate many more free electrons, which increases the conductivity in the order of NiFe LDH, NiFeCr LDH, and NiFeCrCo LDH. In addition, the electrochemically active surface area (ECSA) can be used to characterize the intrinsic catalytic activity of the catalyst; a larger area indicates that more active sites are exposed and can participate in the reaction. The double-layer capacitance (C_dl_) is proportional to the ECSA, and NiFeCrCo LDH has the largest C_dl_ value of 5.8 mF cm^−2^, indicating that it has the largest ECSA (Figure 3f). The smallest overpotential, the smallest Tafel slope, the smallest R_ct_, and the largest C_dl_ all indicate that NiFeCrCo LDH has the best electrocatalytic performance. This is because the introduction of Cr and Co increases the surface area of the material, restructures the electronic structure, and improves the conductivity of the material while ensuring the structural integrity of the material. In addition, synergies between multiple metals can introduce new active sites in the catalyst, and several factors combine to make NiFeCrCo LDH the most appropriate catalyst for water splitting. Another important criterion for evaluating catalysts is their stability. The long-term stability test of NiFeCrCo LDH is shown in Figure 3g and Figure S11. The results show that the initial LSV curve in alkaline solution is not much different from the result after 2000 cycles, except that an oxidation peak is generated at low voltage. The I-t curve shows that NiFeCrCo LDH can run stably for a long time when the current density is 10 mA cm^−2^. The OER can run stably for 100 h, and the HER can run stably for more than 80 h. These results demonstrate the outstanding stability of NiFeCrCo LDH.

### 2.3. OWS Performance

**Figure 4:**
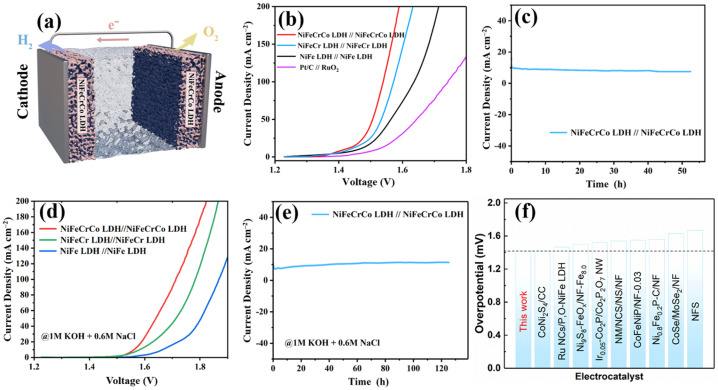
(**a**) Schematic diagram of OWS with two electrodes. (**b**) OWS LSV curves of NiFe LDH//NiFe LDH, NiFeCr LDH//NiFeCr LDH, NiFeCrCo LDH//NiFeCrCo LDH, and Pt/C//RuO_2_ in 1 M KOH, (**c**) I-t curve of OWS at 10 mA cm^−2^ for NiFeCrCo LDH//NiFeCrCo LDH in 1 M KOH, (**d**) OWS LSV curves of NiFe LDH//NiFe LDH, NiFeCr LDH//NiFeCr LDH, and NiFeCrCo LDH//NiFeCrCo LDH in simulated seawater, (**e**) I-t curve of OWS at 10 mA cm^−2^ for NiFeCrCo LDH//NiFeCrCo LDH in simulated seawater, (**f**) comparison of overpotentials of NiFeCrCo LDH and other reported catalysts at a current density of 10 mA cm^−2^ in alkaline solution [46,47,48,49,50,51,52,53,54].

NiFeCrCo LDH exhibited satisfactory electrocatalytic activity in 1 M KOH and is expected to be applied commercially in the future. To evaluate its application potential, a two-electrode electrolysis device was constructed in 1 M KOH with both anode and cathode catalysts of NiFeCrCo LDH (denoted as NiFeCrCo LDH//NiFeCrCo LDH), as shown in Figure 4a. For comparison, other LDH and precious metal catalysts were also tested using the two-electrode electrolysis device. The OWS activity of NiFeCrCo LDH//NiFeCrCo LDH is the highest: the voltage required to reach 10 mA cm^−2^ is only 1.42 V, and reaching 100 mA cm^−2^ requires 1.54 V (Figure 4b). This is significantly better than other LDHs and precious metals such as Pt/C//RuO_2_, even at high current density (Figure S12a). The stability of the NiFeCrCo LDH//NiFeCrCo LDH device in 1 M KOH was investigated. The I-t curve did not show significant damping over 50 h of uninterrupted operation, indicating that this LDH was stable during long-term operation (Figure 4c). The H_2_ and O_2_ yields under OWS conditions (at 1.6 V) were tested using the drainage method (Figures S13 and S14). The results show that the yield ratio of H_2_ to O_2_ is close to 2:1, and the Faradaic efficiency is 97 ± 4%.

In addition, the catalytic activity of the NiFeCrCo LDH in simulated seawater was tested. The OER curve showed that the activity of NiFeCrCo LDH was significantly better than that of NiFe LDH, requiring only 1.44 V to reach 10 mA cm^−2^, which is slightly higher than the overpotential under alkaline conditions (Figure S12b). A comparison of the OWS activity in simulated seawater is shown in Figure 4d, and NiFeCrCo LDH//NiFeCrCo LDH is still the catalyst with the best performance, requiring only 1.57 V to reach 10 mA cm^−2^ and 1.71 V to reach 100 mA cm^−2^. The I-t curve shows that the NiFeCrCo LDH//NiFeCrCo LDH device can operate stably in simulated seawater for more than 120 h (Figure 4e). The recently reported electrocatalytic performances of state-of-the-art OWS catalysts in alkaline and simulated seawater is compared in Figure 4f and Table S1 [47,48,49,50,51,52,53,54,55]. Compared with most catalysts, NiFeCrCo LDH has excellent OWS performance as a bifunctional electrocatalyst in both alkaline and simulated seawater, thus meeting the requirements for long-term operation. Although NiFeCrCo LDH demonstrates outstanding OWS performance, examining the complex lattice featuring a random distribution of metal elements remains challenging. Revealing the local and macro-scale statistics of the distribution of metal elements within the lattice is essential for understanding the precise entropy and active sites of these materials.

## 3. Materials and Methods

NNiFeCrCo LDH was synthesized using a facile one-step hydrothermal method. The synthesis details are as follows: Ni(NO_3_)_2_·6H_2_O (0.5 mmol), Fe(NO_3_)_3_·9H_2_O (0.25 mmol), Cr(NO_3_)_3_·9H_2_O (0.25 mmol), Co(NO_3_)_2_·6H_2_O (0.5 mmol), CO(NH_2_)_2_ (5 mmol), and NH_4_F (5 mmol) were dissolved in 36 mL of distilled water and stirred gently until the solution became clear. Then, the solution was transferred to a 40 mL stainless steel autoclave (Teflon-lined) containing NF and sealed. The sample was kept in a drying oven at 120 °C for 12 h and then cooled naturally to room temperature. Subsequently, the NF grown with NiFe LDH was removed, rinsed with distilled water and ethanol several times to remove the residue, and finally dried in a vacuum oven at 80 °C for 6 h.

Details of the synthesis, characterization, and electrochemical testing of LDH can be found in the Supporting Information.

## 4. Conclusions

In summary, NiFe LDH, NiFeCr LDH, and NiFeCrCo LDH were synthesized using a one-step hydrothermal method. The OER, HER, and OWS performances improved with the introduction of additional element types. The performance followed the order NiFe LDH < NiFeCr LDH < NiFeCrCo LDH. NiFeCrCo LDH exhibited the best performance, with excellent OWS activity and stability. Under alkaline conditions, NiFeCrCo LDH required only 1.42 V to reach a current density of 10 mA cm^−2^ and 1.54 V to reach 100 mA cm^−2^. Under simulated seawater conditions, only 1.57 V was required to reach 10 mA cm^−2^ and 1.71 V to reach 100 mA cm^−2^. The results indicated that Cr donated electrons to Fe, and the introduction of Co induced Cr to donate more electrons to Fe. This is because Cr has the lowest electronegativity, resulting in special electronic states in NiFeCrCo LDH. These special electronic states were also attributed to the increase in entropy following the order NiFe LDH < NiFeCr LDH < NiFeCrCo LDH, which modulated the electronic structure. It is concluded that entropy-increase engineering could optimize the electronic structure of NiFeCrCo LDH and enhance the performance of OER, HER, and OWS. This work provides a new perspective on utilizing entropy effects in LDH as high-performance OWS catalysts.

## Data Availability

All the relevant data are included in this published article.

## Supplementary Materials

The following supporting information can be downloaded at: https://www.mdpi.com/article/10.3390/molecules30071461/s1, Figure S1: XRD of NiFeCrCo LDH on NF prepared by one-step hydrothermal method; Figure S2: (a) TEM, (b) HRTEM, (c) the IFFT graph of the yellow box in (b), (d) the line-scanning intensity profile of the red dotted box in (c), (e–e3) STEM-EDS mapping of NiFe LDH; Figure S3: (a) TEM, (b) HRTEM, (c) the IFFT graph of the blue box in (b), (d) the line-scanning intensity profile of the pink dotted box in (c), (e–e4) STEM-EDS mapping of NiFeCr LDH; Figure S4: (a) TEM, (b) HRTEM, (c) the IFFT graph of the orange box in (b), (d) the line-scanning intensity profile of the purple dotted box in (c) of NiFeCrCo LDH; Figure S5: The schematic diagram of NiFe LDH, NiFeCr LDH, and NiFeCrCo LDH. The atomic distribution in the structures are shows that in NiFeCr LDH, Cr atoms substitute Fe atoms with a Fe:Cr ratio of 1:1 and are randomly distributed; in NiFeCrCo LDH, Cr atoms substitute Fe atoms with a Fe:Cr ratio of 1:1, Co atoms substitute Ni atoms with a Ni:Co ratio of 1:1 and are randomly distributed; Figure S6: (a) Linear sweep voltammetry (LSV) curves of HER for different ternary LDH are formed by mixing different divalent metal ions with NiFe LDH in 1 M KOH solution, (b) LSV curves of HER for different ternary LDH are formed by mixing different trivalent metal ions with NiFe LDH in 1 M KOH solution, (c) LSV curves of OER for different ternary LDH are formed by mixing different divalent metal ions with NiFe LDH in 1 M KOH solution, (d) LSV curves of OER for different ternary LDH are formed by mixing different trivalent metal ions with NiFe LDH in 1 M KOH solution; Figure S7: (a) LSV curves of HER for different quaternary LDH are formed by mixing different divalent metal ions with NiFeCr LDH in 1 M KOH solution, (b) LSV curves of HER for different quaternary LDH are formed by mixing different trivalent metal ions with NiFeCr LDH in 1 M KOH solution, (c) LSV curves of OER for different quaternary LDH are formed by mixing different divalent metal ions with NiFeCr LDH in 1 M KOH solution, (d) LSV curves of OER for different quaternary LDH are formed by mixing different trivalent metal ions with NiFeCr LDH in 1 M KOH solution; Figure S8: LSV curves of OER for different LDH and RuO_2_ at high potentials; Figure S9: LSV curves of HER for different LDH and Pt/C at (a) low potentials and (b) high potentials, (c) the overpotential of the catalysts with different current density (the value of * is approximate); Figure S10: (a) TOF values of NiFeCrCo LDH, NiFeCr LDH and NiFe LDH at different overpotentials, (b) the corresponding TOF values at overpotential of 300 mV; Figure S11: I-t curve of HER at 10 mA cm^−2^ for NiFeCrCo LDH; Figure S12: (a) LSV curves of different LDH for OWS at high potentials in 1M KOH, (b) LSV curves of NiFe LDH and NiFeCrCo LDH for OER in simulated seawater; Figure S13: (a) Optical photo of gas collection set-up of the H-type electrolytic cell, (b) H_2_/O_2_ gas collected in a measuring jar at every ~600 s interval at 1.6 V; Figure S14: (a) Faradaic efficiency of hydrogen evolution and oxygen evolution during OWS for NiFeCrCo LDH at 1.6 V for 4000 s. The blue and pink balls represent the molar amounts of H_2_ and O_2_ collected by the drainage method every 600 s. The blue and pink lines indicate the theoretical molar amounts of H_2_ and O_2_ produced. The yield ratio of H_2_ to O_2_ is close to 2:1, and (b) the Faradaic efficiency over 97 ± 4%; Table S1: Comparison of overpotential to achieve 10 and 100 mA cm^−2^ for OWS of various kinds of bifunctional electrocatalysts in 1 M KOH solution and simulated seawater. Refs. [56,57,58,59,60,61,62,63,64,65,66,67] are cited in the Supplementary Materials.
