# Supplementary material for: Enhanced the Overall Water Splitting Performance of Quaternary NiFeCrCo LDH: Via Increasing Entropy
Source: Molecules. 2025 Mar 25;30(7):1461. doi: 10.3390/molecules30071461 (PMC11990446; doi:10.3390/molecules30071461)
Supplement: Supplementary file 1 [file molecules-30-01461-s001.zip › molecules-3463597-supplementary.pdf]

Supporting Information

Enhanced the Overall Water Splitting Performance of Quaternary NiFeCrCo LDH: Via Increasing Entropy

Xin Liu [1], Li Bai [1], Xinrong Guo [1], Haoyu Li [1], Xiaoyan Liu [1,2], Jian Cao [1,2], Lili Yang [1,2], Maobin Wei [1,2], Yanli Chen [1,2,*], Huilian Liu [1,2,*] and Qiang Tao [3]

1 Key Laboratory of Functional Materials Physics and Chemistry of the Ministry of Education, Jilin Normal University, Changchun 130103, China; 15330783861@163.com (X.L.); 15326575020@163.com (L.B.); 17335887075@163.com (X.G.); 15568559566@163.com (H.L.); liuxiaoyan1437@163.com (X.L.); caojian_928@163.com (J.C.); llyang1980@126.com (L.Y.); jlsdzccw@126.com (M.W.)

2 National Demonstration Center for Experimental Physics Education, Jilin Normal University, Siping 136000, China

3 Synergetic Extreme Condition High-Pressure Science Center, State Key Laboratory of Superhard Materials, College of Physics, Jilin University, Changchun 130012, China; qiangtao@jlu.edu.cn

* Correspondence: ylchen@jlnu.edu.cn (Y.C.); lhl541@126.com (H.L.)

Keywords: NiFeCrCo LDH, one-step hydrothermal method, entropy, electrocatalyst, overall water splitting.

Experimental Section

Chemicals

Cobalt nitrate ($Co(NO_3)_2 \cdot 6H_2O$, ≥99.9 %), Iron nitrate ($Fe(NO_3)_3 \cdot 9H_2O$, ≥99.99 %), Nickel nitrate ($Ni(NO_3)_2 \cdot 6H_2O$, ≥98 %), Chromium nitrate ($Cr(NO_3)_3 \cdot 9H_2O$, ≥99 %), Copper nitrate ($Cu(NO_3)_2 \cdot 3H_2O$, ≥99 %), Zinc nitrate ($Zn(NO_3)_2 \cdot 6H_2O$, ≥99 %),

Manganese nitrate ($Mn(NO_3)_2·xH_2O$, ≥98 %), Aluminum nitrate ($Al(NO_3)_3·9H_2O$, ≥99.9 %), nitrate ($Co(NO_3)_2·6H_2O$, ≥99.9 %), and Iridium oxide (IrO_2, ≥99.9 %) were purchased from Aladdin. Vanadium chloride (VCl_3, 99 %) and Pt/C (20 wt%) were purchased from Macklin. It bought hydrochloric acid (HCl, 37 %), potassium hydroxide (KOH, ≥99 %), Sodium chloride (NaCl, ≥99.5 %), Ammonium fluoride (NH_4F, ≥96 %), acetone (C_3H_6O, ≥99 %), ethanol (C_2H_6O, ≥99.5 %) and urea ($CO(NH_2)_2$, 99 %) from Sinopharm Chemical Reagent Co. Ltd. All chemicals were used directly without further purification. Nickel foam (NF) was obtained from Kunshan Guangjiayuan New Material Co. Ltd.

Before electrodeposition, NF was processed by 1.0 M HCl solution, acetone, deionized water and ethanol. Ultrasonic was continued in each solvent for 10 minutes to achieve the purpose of cleaning the surface. Then it was vacuum-dried at 60 °C for 12 hours.

Synthesis of NiFe LDH

NiFe LDH was synthesized via a facile one-step hydrothermal method. $Ni(NO_3)_2·6H_2O$ (1 mmol), $Fe(NO_3)_3·9H_2O$ (0.5 mmol), $CO(NH_2)_2$ (5 mmol), and NH_4F (5 mmol) were dissolved in 36 mL distilled water, stirred gently until the solution is clarified. Then, the solution was transferred to a 40 mL stainless-steel autoclave (Teflon-lined) containing NF and sealed. Keep in a drying oven at 120 °C for 12 hours. And then cooled naturally to room temperature. Subsequently, the NF grewn with NiFe LDH was taken out, rinsed with distilled water and ethanol several times to remove the residue, and finally dried in a vacuum oven at 80 °C for 6 h.

Synthesis of Ternary NiFeM LDH

The synthesis method of Ternary NiFeM LDH is similar to that of NiFe LDH, except that the proportion of raw materials was adjusted. If +3 valent metal ions were added, the total amount of $Fe(NO_3)_3·9H_2O$ and $M(NO_3)_3$ is 0.5 mmol, and the metal molar ratio is Fe:M = 1:1; If +2 valent metal ions were added, the total amount of

$Ni(NO_3)_2{\cdot}6H_2O$ and $N(NO_3)_2$ is 1 mmol, and the metal molar ratio is Ni:N = 1:1. For instance, NiFeCr LDH: $Ni(NO_3)_2{\cdot}6H_2O$ (1 mmol), $Fe(NO_3)_3{\cdot}9H_2O$ (0.25 mmol), $Cr(NO_3)_3{\cdot}9H_2O$ (0.25 mmol), $CO(NH_2)_2$ (5 mmol), and NH_4F (5 mmol) were dissolved in 36 mL distilled water, stirred gently until the solution is clarified. Then, the solution was transferred to a 40 mL stainless-steel autoclave (Teflon-lined) containing NF and sealed. Keep in a drying oven at 120 °C for 12 hours. And then cooled naturally to room temperature. Subsequently, the NF grewn with NiFe LDH was taken out, rinsed with distilled water and ethanol several times to remove the residue, and finally dried in a vacuum oven at 80 °C for 6 h.

Synthesis of quaternary NiFeMN LDH

The synthesis method of of quaternary NiFeMN LDH was the same as above. Similarly, when the two introduced metal ions are both +3 valent states, the total amount of $Fe(NO_3)_3{\cdot}9H_2O$, $M(NO_3)_3$, and $M'(NO_3)_3$ is 0.5 mmol, the metal molar ratio is Fe:M:M' = 1:1:1; when the two introduced metal ions are both +2 valent states, the total amount of $Ni(NO_3)_2{\cdot}6H_2O$, $N(NO_3)_3$, and $N'(NO_3)_3$ is 1 mmol, the metal molar ratio is Ni:N:N' = 1:1:1; when two metal ions are introduced, one is +2 valent states and the other is +3 valent states, the total amount of $Fe(NO_3)_3{\cdot}9H_2O$ and $M(NO_3)_3$ is 0.5 mmol, the metal molar ratio is Fe:M = 1:1, and the total amount of $Ni(NO_3)_2{\cdot}6H_2O$ and $N(NO_3)_2$ is 1 mmol, the metal molar ratio is Ni:N = 1:1.

Preparation of catalyst electrode.

Preparation of the Pt/C/NF electrocatalyst: Pt/C (2 mg 20 wt%) was put into 200 μL lsopropyl alcohol for 25 minutes of ultrasound to make it uniformly dispersed. The obtained 2 μL dispersion was drop-casting onto NF. Finally, the samples were coated by 2 μL of 0.3 % Nafion solution and letting to dry.

Preparation of the RuO_2/NF electrocatalyst: RuO_2 (2 mg) was put into 200 μL lsopropyl alcohol for 25 minutes of ultrasound to make it uniformly dispersed. The obtained 2 μL dispersion was drop-casting onto NF. Finally, the samples were coated

by 2 μL of 0.3% Nafion solution and letting to dry.

Characterizations

The morphology and elements information of different LDH were probed by Field-emission scanning electron microscope (FESEM, HITACHI S-4800). The high-resolution transmission electron microscope (TEM) images were obtained with a JEM-2200FS TEM operating at 200 KV. The structures were tested by X-ray diffraction (XRD, Rigaku D/Max-ga, Cu Ka radiation). The compositions were recorded by X-ray photoelectron spectroscopy (XPS, ESCALAB 250Xi, Thermal Fisher). The Brunauer-Emmett- Teller (BET) surface area analysis was conducted on ASAP 2020HD88 instrument.

Electrochemical measurements

The electrochemical measurements were performed by CH Instrument (Model 760E) using a standard three-electrode system in 1 M KOH solution. Above prepared catalysts were directly used as the working electrode. Hg/HgO electrode and carbon rod were used as reference electrode and counter electrode, respectively. The overpotential is expressed as the value versus the reversible hydrogen electrode (RHE) in this work. According to the Nernst equation given by $E_{vs.RHE} = E_{Hg/HgO} + 0.098\ V + 0.059 \times pH$. Linear sweep voltammetry (LSV) curves towards HER and OER were obtained by 1.0 M KOH electrolyte solution at a scan rate of 5 mV s^{-1}. The current density is normalized by a geometric area (3 mm × 3 mm) unless specified. Electrochemical impedance spectroscopy (EIS) was measured from $1-10^5$ Hz with AC amplitude of 5 mV at the overpotentials of 500 mV for OER. The electrochemical active surface area (ECSA) was measured by cyclic voltammetry (CV) with different scans in non-faradaic potential region (4, 8, 12, 16 and 20 mV s^{-1}). The ECSA of the sample can be calculated by dividing C_{dl} by the specific capacitance (C_s) of the material (ECSA = C_{dl}/C_s). A C_s value of 0.04 mF cm^{-2} was used herein [1]. The turnover frequency (TOF) was

calculated as described in previous reports [2]. The longtime stability is evaluated by chronoamperometric measurements. The performance of overall water spitting (OWS) in alkaline conditions and simulated seawater were tested in a standard two-electrode system (1.0 M KOH and 1.0 M KOH + 0.6 M NaCl). During OWS testing, NiFeCrCo LDH are used as both the anode and cathode, which the counter electrode and the reference electrode in the electrochemical workstation were connected at the cathode, and the working electrode was connected at the anode.

The Faradic efficiency during OWS is defined as the ratio of the amount of experimentally determined H_2/O_2 to that of the theoretically expected H_2/O_2 from the HER/OER. Accordingly, we collected the hydrogen gas and oxygen gas evolved from the water splitting reaction by the water drainage method. The test was performed with a two-electrode system in an H-type electrolytic cell at room temperature (~23 °C) (Fig S13a). The instrument used to apply voltage was a CH Instrument electrochemical workstation (Model 760E). The electrolyte volume was 35 mL, the headspace of the electrochemical cell was 15 mL. The headspace volume before the test was 0 mL, because the measuring cylinder was filled with solution at first. The geometric surface area of the electrodes was 0.09 cm^2. Before the test, the electrolyte was purged with N_2 for more than 30 min, the bubbles of the formed H_2/O_2 slowly stirred the electrolyte. The test was conducted at a constant voltage of 1.6 V in 1 M KOH electrolyte. Then, we calculated the moles of H_2/O_2 generated from the reaction based on the ideal gas law, $N = i \times t /(n \times F)$, where N is the theoretical amount (mol) of gaseous products, i is the current (A), t is the time (s), n is the number of electrons transferred (2 electrons for HER, 4 electrons for OER), and F is the Faraday constant. As for the theoretical value, we assumed that there was 100 % current efficiency during the reaction, which means only the HER and OER processes were occurring at the working electrode and counter electrode.

Entropy of LDH

The S_{conf} of bulk materials ($S_{conf(b)}$) is

$$S_{conf(b)} = -R\sum_{i}^{N} x_i ln x_i$$

where R represents the ideal gas constant, and x_i denotes the mole fraction of component i [3]. This can reflect the entropy of grain inner for nano-sized materials. But due to the surface effect, the correction term of surface ($S_{surface}$) should be added. Therefore, the S_{conf} of nano-sized materials ($S_{conf(n)}$) can be written as:

$$S_{conf(n)} = S_{conf(b)} + S_{surface} = -R\sum_{i}^{N} x_i ln x_i + S_{surface}$$

The $S_{surface}$ is dominated by the surface effect. If we can assume that the surface effect is only determined by the size of the nano-layer and is independent of different elements. Then we can estimate that the surface effect is related to the ratio of surface atoms to total atoms, that is, it is related to the proportion of the number of surface atoms and inner atoms. If the number of surface atoms is N_s, the number of inner atoms is N_b, and the number of total atoms is N, then there is a relation $N = N_b + N_s$. Since the samples are nano-layers, we can ignore the surface atoms on the edge sides. For N_s, it represents the atoms of only two layers. Thus, $N_s = 2S/r^2$, where S is the surface area of the basal plane of one layer, r is the one-dimension size, and r^2 is the surface area of one atom. By the way, the number of inner atoms is $N_b = \frac{S(n-2)r}{r^3} = \frac{S(n-2)}{r^2}$, where n is the number of layers.

We can then estimate that $\frac{N_s}{N} = \frac{2}{n}$, $\frac{N_b}{N} = \frac{n-2}{n}$. If the thickness of nano-layers is l, the interlayer spacing is d, and $n = \frac{l+d}{d}$, then $\frac{N_s}{N} = \frac{2d}{l+d}$, $\frac{N_b}{N} = \frac{l-d}{l+d}$. So, $S_{conf(b)}$, and $S_{surface}$ can be estimated by:

$$S_{conf(b)} = -R\sum_{i}^{N} \frac{N_b}{N} x_i ln \frac{N_b}{N} x_i = -R\sum_{i}^{N} \frac{l-d}{l+d} x_i ln \frac{l-d}{l+d} x_i$$

$$S_{surface} = -RK\sum_{i}^{N} \frac{N_s}{N} x_i ln \frac{N_s}{N} x_i = -RK\sum_{i}^{N} \frac{2d}{l+d} x_i ln \frac{2d}{l+d} x_i$$

$$S_{conf(n)} = S_{conf(b)} + S_{surface}$$

Where K is the correction factor, which depends on different materials.

The thicknesses (l) of NiFe LDH, NiFeCr LDH, and NiFeCrCo LDH are 116.8 nm, 62.6 nm, and 51.5 nm, respectively. The interlayer spacing (d) of LDH is 0.7749 nm. The configurational entropies ($S_{conf(n)}$) of NiFe LDH, NiFeCr LDH, and NiFeCrCo LDH are $(0.641 + 0.065K)R$, $(0.778 + 0.085K)R$, $(1.319 + 0.143K)R$, respectively. Since all of the samples are LDH, the values of K for all the samples are close. Therefore, it can be concluded that $S_{conf(n)}$ increases in the order of NiFe LDH, NiFeCr LDH, and NiFeCrCo LDH.

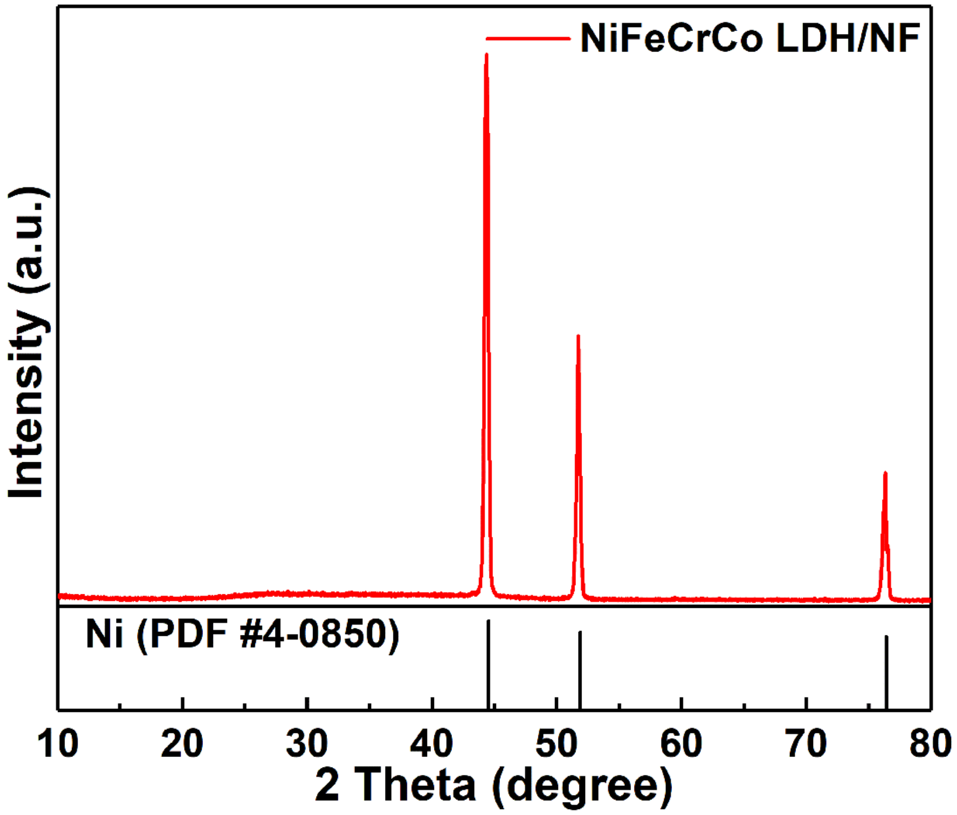

Figure S1. XRD of NiFeCrCo LDH on NF prepared by one-step hydrothermal method.

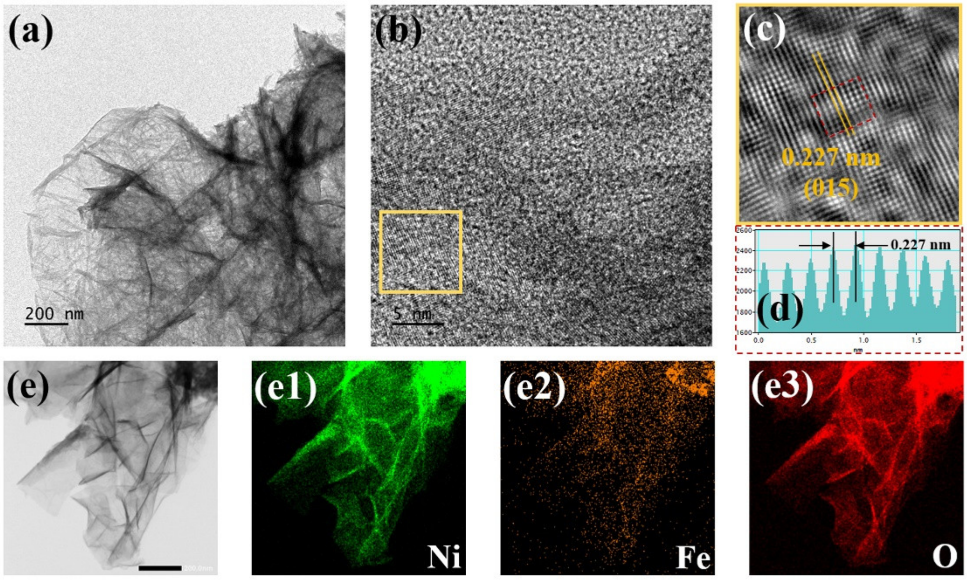

Figure S2. (**a**) TEM, (**b**) HRTEM, (**c**) the IFFT graph of the yellow box in (**b**), (**d**) the line-scanning intensity profile of the red dotted box in (**c**), (**e–e3**) STEM-EDS mapping of NiFe LDH.

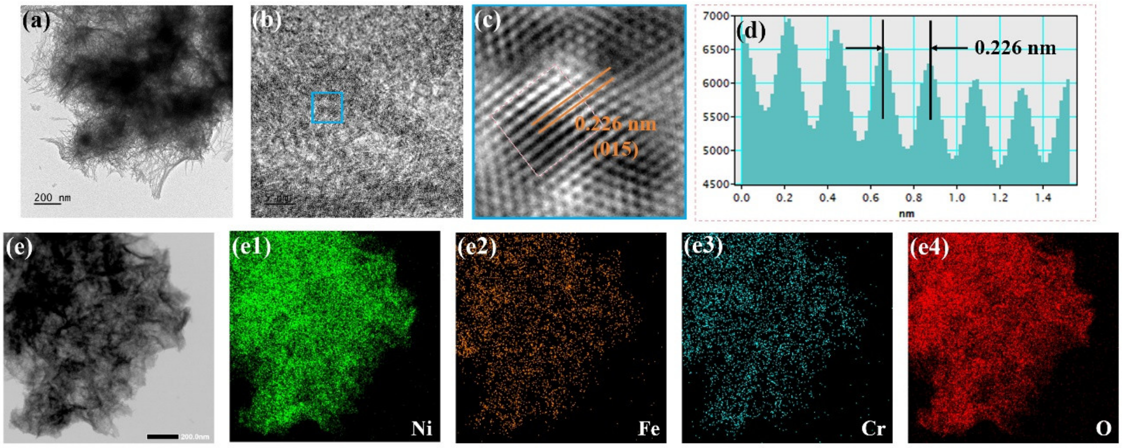

Figure S3. (**a**) TEM, (**b**) HRTEM, (**c**) the IFFT graph of the blue box in (**b**), (**d**) the line-scanning intensity profile of the pink dotted box in (**c**), (**e–e4**) STEM-EDS mapping of NiFeCr LDH.

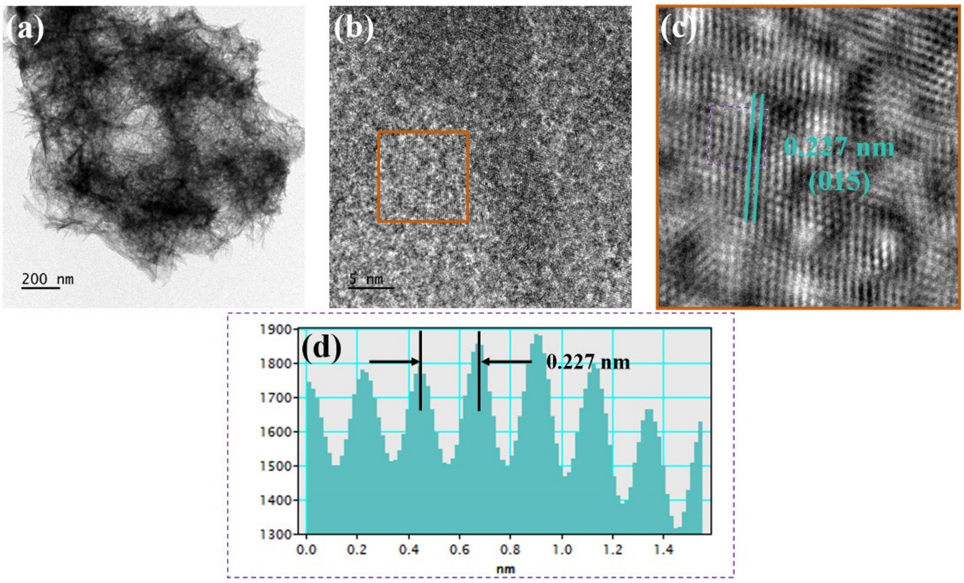

Figure S4. (**a**) TEM, (**b**) HRTEM, (**c**) the IFFT graph of the orange box in (**b**), (**d**) the line-scanning intensity profile of the purple dotted box in (**c**) of NiFeCrCo LDH.

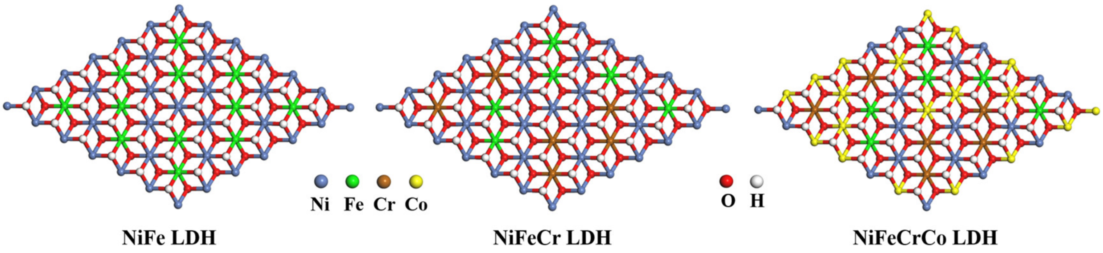

Figure S5. The schematic diagram of NiFe LDH, NiFeCr LDH, and NiFeCrCo LDH. The atomic distribution in the structures are shows that in NiFeCr LDH, Cr atoms substitute Fe atoms with a Fe:Cr ratio of 1:1 and are randomly distributed; in NiFeCrCo LDH, Cr atoms substitute Fe atoms with a Fe:Cr ratio of 1:1, Co atoms substitute Ni atoms with a Ni:Co ratio of 1:1 and are randomly distributed.

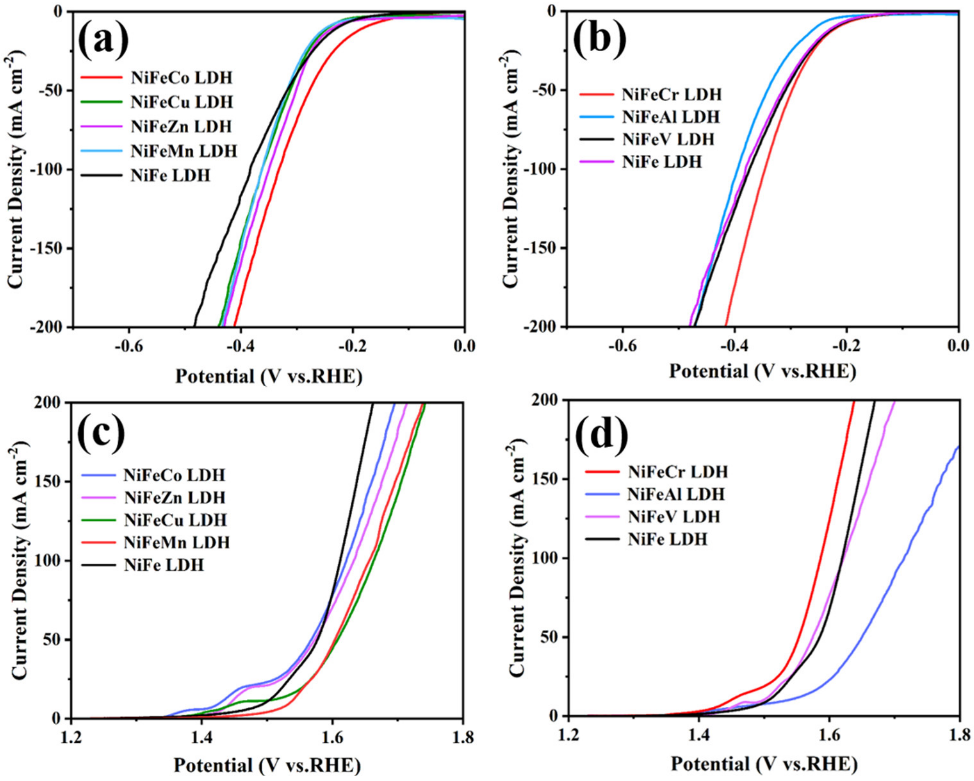

Figure S6. (**a**) Linear sweep voltammetry (LSV) curves of HER for different ternary LDH are formed by mixing different divalent metal ions with NiFe LDH in 1 M KOH solution, (**b**) LSV curves of HER for different ternary LDH are formed by mixing different trivalent metal ions with NiFe LDH in 1 M KOH solution, (**c**) LSV curves of OER for different ternary LDH are formed by mixing different divalent metal ions with NiFe LDH in 1 M KOH solution, (**d**) LSV curves of OER for different ternary LDH are formed by mixing different trivalent metal ions with NiFe LDH in 1 M KOH solution.

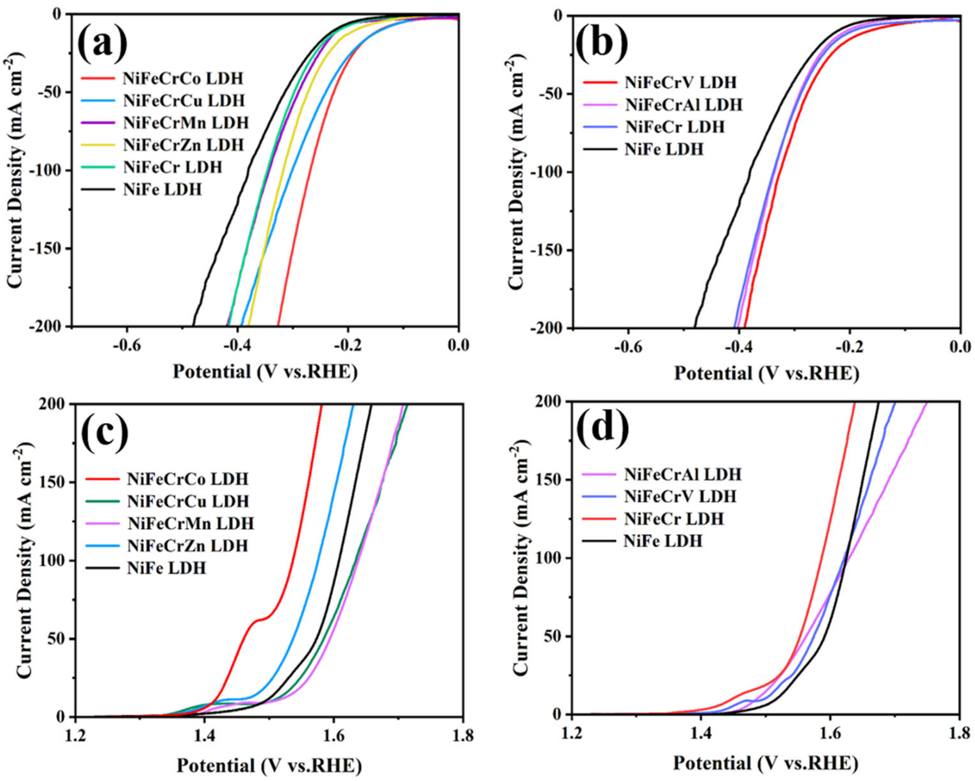

Figure S7. (**a**) LSV curves of HER for different quaternary LDH are formed by mixing different divalent metal ions with NiFeCr LDH in 1 M KOH solution, (**b**) LSV curves of HER for different quaternary LDH are formed by mixing different trivalent metal ions with NiFeCr LDH in 1 M KOH solution, (**c**) LSV curves of OER for different quaternary LDH are formed by mixing different divalent metal ions with NiFeCr LDH in 1 M KOH solution, (**d**) LSV curves of OER for different quaternary LDH are formed by mixing different trivalent metal ions with NiFeCr LDH in 1 M KOH solution.

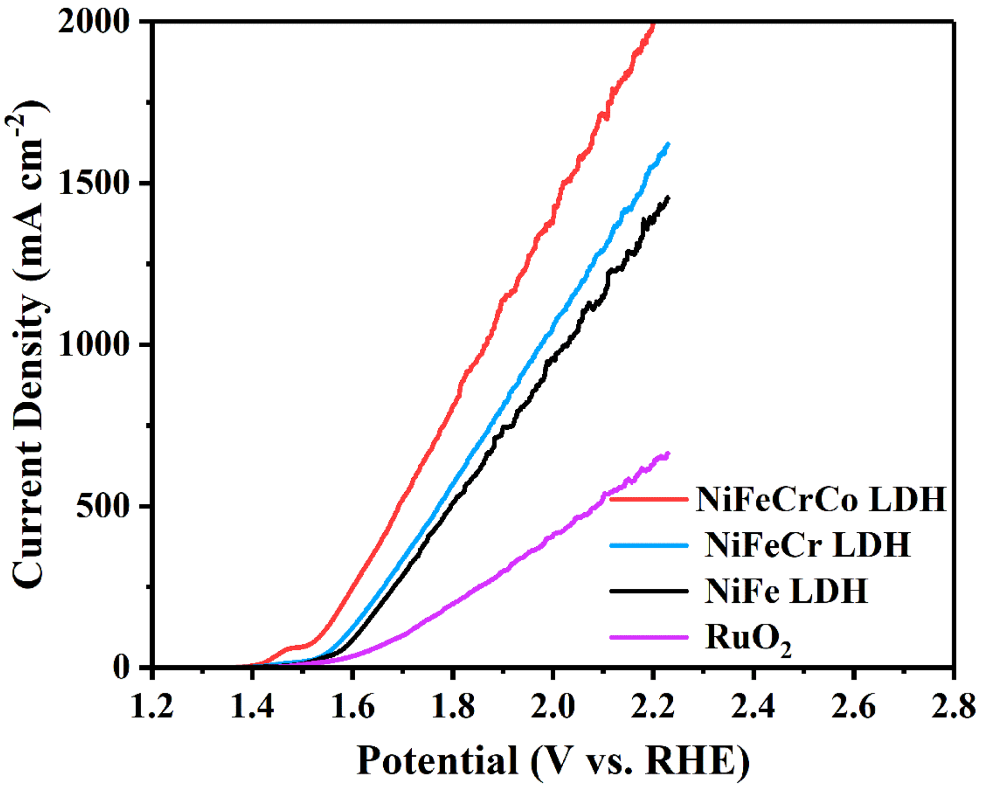

Figure S8. LSV curves of OER for different LDH and RuO_2 at high potentials.

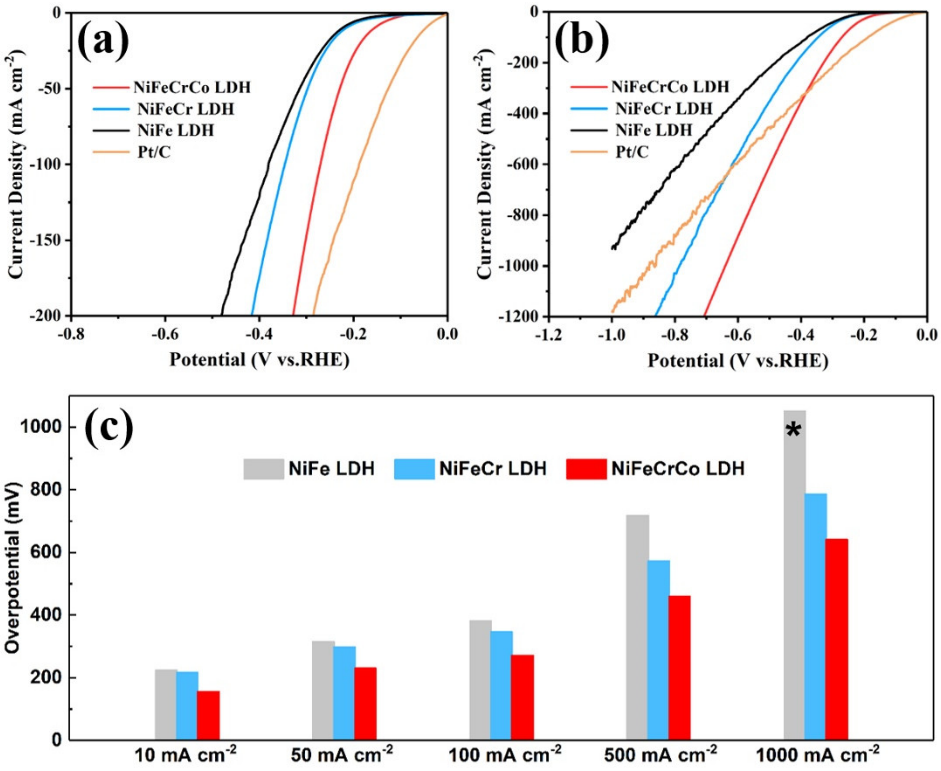

Figure S9. LSV curves of HER for different LDH and Pt/C at (**a**) low potentials and (**b**) high potentials, (**c**) the overpotential of the catalysts with different current density (the value of * is approximate).

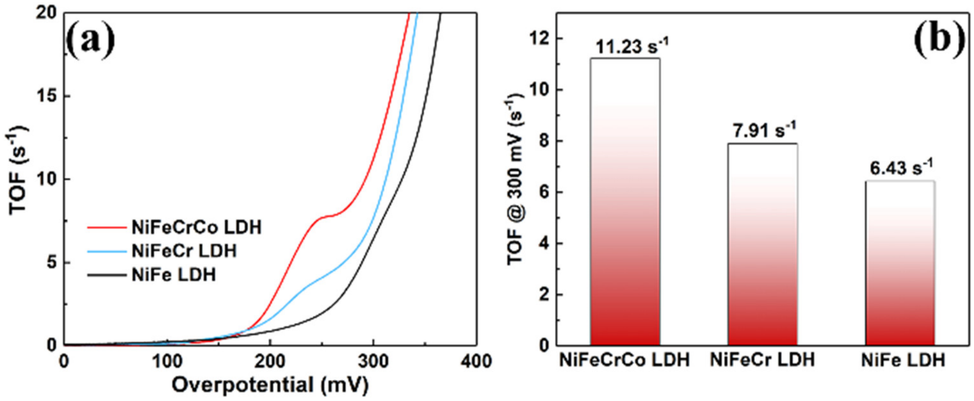

Figure S10. (**a**) TOF values of NiFeCrCo LDH, NiFeCr LDH and NiFe LDH at different overpotentials, (**b**) the corresponding TOF values at overpotential of 300 mV.

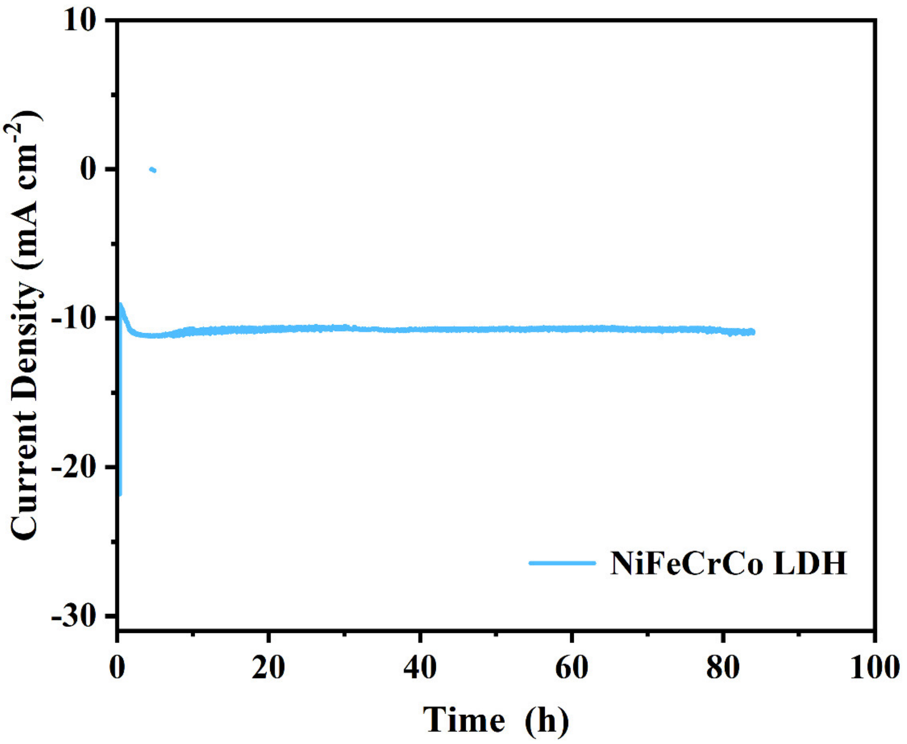

Figure S11. I-t curve of HER at 10 mA cm^{-2} for NiFeCrCo LDH.

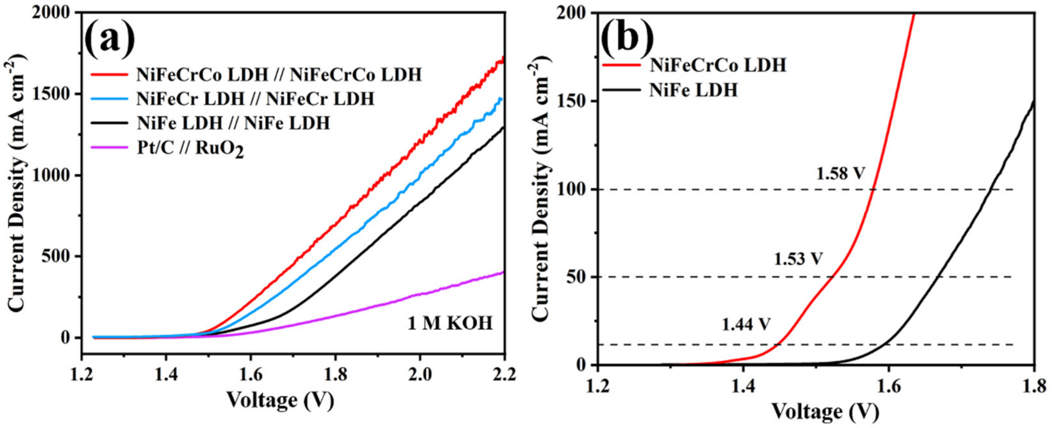

Figure S12. (**a**) LSV curves of different LDH for OWS at high potentials in 1M KOH, (**b**) LSV curves of NiFe LDH and NiFeCrCo LDH for OER in simulated seawater.

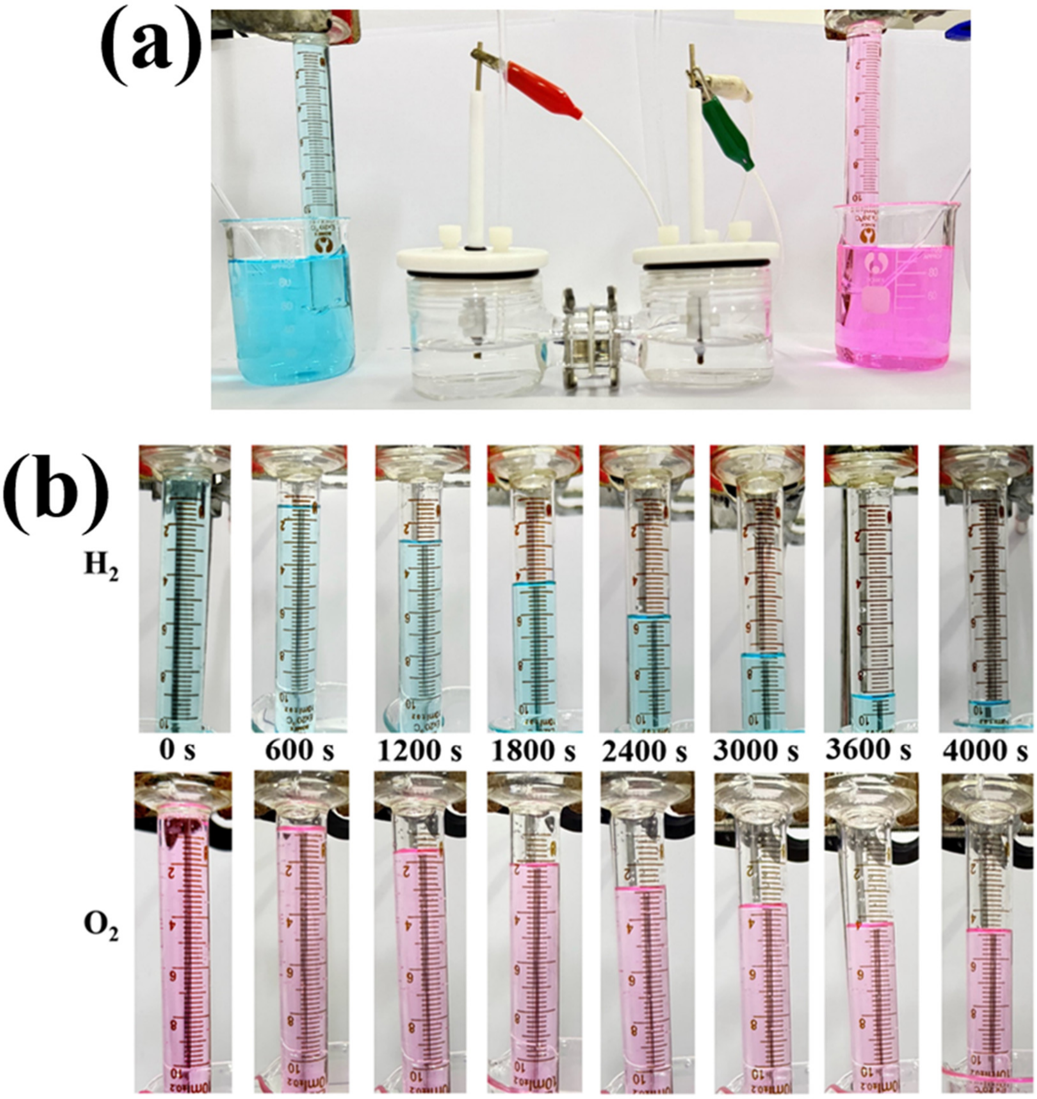

Figure S13. (**a**) Optical photo of gas collection set-up of the H-type electrolytic cell, (**b**) H_2/O_2 gas collected in a measuring jar at every ~600 s interval at 1.6 V.

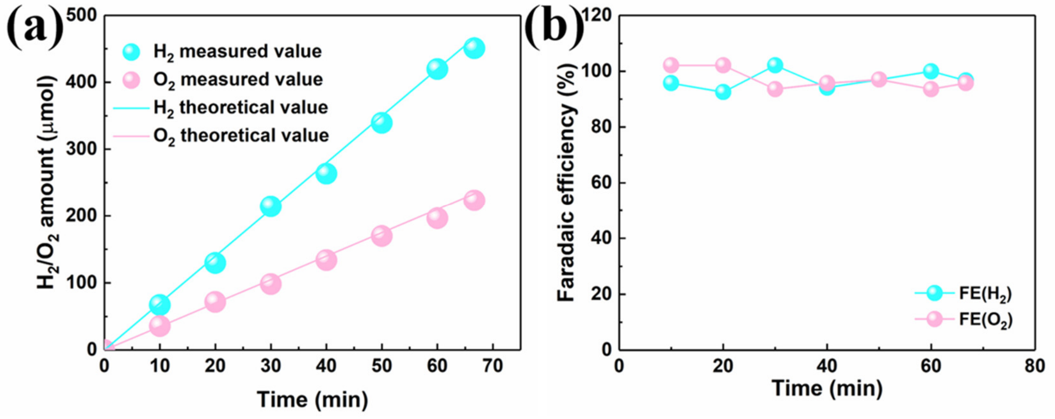

Figure S14. (**a**) Faradaic efficiency of hydrogen evolution and oxygen evolution during OWS for NiFeCrCo LDH at 1.6 V for 4000 s. The blue and pink balls represent the molar amounts of H_2 and O_2 collected by the drainage method every 600 s. The blue and pink lines indicate the theoretical molar amounts of H_2 and O_2 produced. The yield ratio of H_2 to O_2 is close to 2:1, and (**b**) the Faradaic efficiency over 97 ± 4 %.

Table S1. Comparison of overpotential to achieve 10 and 100 mA cm^{-2} for OWS of various kinds of bifunctional electrocatalysts in 1 M KOH solution and simulated seawater.

Electrocatalyst	Electrolytes	η_{10} (V)	η_{100} (V)	References
NiFeCrCo LDH	1 M KOH	1.42	1.54	This work
	simulated seawater	1.57	1.71	
Ni_9S_8-FeO_x/NF-$Fe_{8.0}$	1 M KOH	1.501	1.707	[4]
	simulated seawater	1.578	1.794	
CoFeNiP/NF-0.03	1 M KOH	1.55	1.675	[5]
	simulated seawater	1.48	1.66	
NiFe@Ni	simulated seawater	-	1.73	[6]
NF@MoSCo/Co_2P	1 M KOH	-	1.59	[7]
	simulated seawater	-	1.61	
(Ni-Ru-Mo)P@F-CDS	1 M KOH	-	1.508	[8]
	simulated seawater	-	1.564	
NFS	1 M KOH	1.67	-	[9]
	simulated seawater	1.84	-	
NiCoP@HP	simulated seawater	1.44	-	[10]
NiMoSe@CC	simulated seawater	1.63	-	[11]
NM/NCS/NS/NF	1 M KOH	1.54	-	[12]

	simulated seawater	1.56	-	
$CoNi_2S_4$/CC	1 M KOH	1.42	1.86	[13]
	simulated seawater	1.486	2.041	
Co-P@NN	simulated seawater	1.512	-	[14]
$Ni_{0.8}Fe_{0.2}$P-C/NF	1 M KOH	1.56	-	[15]
Ni@NP@NCP(F/SO/H)	1 M KOH	-	1.697	[16]
	simulated seawater	-	1.724	
$Ir_{0.05}$-$Co_2P/Co_2P_2O_7$ NW	1 M KOH	1.52	-	[17]
	simulated seawater	1.62	-	
CoSe/$MoSe_2$/NF	1 M KOH	1.63	-	[18]
	simulated seawater	1.69	-	
NF@NM-NP@NiFe LDH	simulated seawater	1.46	1.7	[19]
S-$NiMoO_4$@NiFe LDH	simulated seawater	1.68	-	[20]
Ru NCs/P, O-NiFe LDH/NF	1 M KOH	1.466	-	[21]
	simulated seawater	1.506	-	

References

1. McCrory, C. C.; Jung, S.; Ferrer, I. M.; Chatman, S. M.; Peters, J. C.; Jaramillo, T. F., Benchmarking hydrogen evolving reaction and oxygen evolving reaction electrocatalysts for solar water splitting devices. *J. Am. Chem. Soc.* **2015,** *137* (13), 4347-4357.

2. Wang, T.; Guo, X.; Zhang, J.; Xi, P.; Peng, S.; Gao, D., Electronic structure modulation of NiS_2 by transition metal doping for accelerating the hydrogen evolution reaction. *J. Mater. Chem. A* **2019,** *7* (9), 4971-4976.

3. Miracle, D. B.; Senkov, O. N., A critical review of high entropy alloys and related concepts. *Acta Mater.* **2017,** *122*, 448-511.

4. Xiao, W.; Chen, Y.; Ke, C.; Han, F.; Wang, C.; Yang, X., Engineering the

Heterostructured Ni_9S_8–FeO_x Nanoarrays for Electrocatalytic (Sea)Water Splitting. *ACS Appl. Nano Mater.* **2025,** *8* (1), 512-523.

5. Ye, R.; Sheng, Z.; Yang, P.; Xu, L.; Tao, Y.; Wu, X.; Cui, X., Ni-doped CoFeP as high-efficeint electrocatalysts for water-splitting. *Electrochim. Acta* **2024,** *507*, 145152.

6. Huang, G.; Wang, Y.; Hao, W.; Lu, W.; Wang, Y.; Huang, Z.; Fan, J., Accurately prepared the large-area and efficiently 3D electrodes for overall seawater splitting. *J. Electroanal. Chem.* **2024,** *973*, 118671.

7. Yang, P.; Liu, B.; Zhang, X.; Li, K.; Hu, D.; Xing, H.; Zhu, Q., Synthesis of nanoflower-like NF@MoSCo/Co_2P composites for overall seawater splitting. *CrystEngComm* **2024,** *26* (41), 5892-5906.

8. Jiang, Y.; Yu, J.; Song, H.; Du, L.; Sun, W.; Cui, Y.; Su, Y.; Sun, M.; Yin, G.; Lu, S., Enhanced water-splitting performance: Interface-engineered tri-metal phosphides with carbon dots modification. *Carbon Energy* **2024,** *6* (10), 631.

9. Zhang, S.; Ji, Y.; Wang, S.; Zhang, P.; Shi, D.; Lu, F.; Zhang, B., Sulfur doping induces internal polarization field in NiFe-LDH for bifunctioanl HER/OER and overall water/simulated seawater splitting. *J. Alloy. Compd.* **2024,** *1002*, 175323.

10. Ma, X.; Liang, R.; Wang, Y.; Wu, L.; Lei, F.; Fan, J.; Wang, L.; Hao, W., Large-area, flexible bimetallic phosphorus-based electrodes for prolong-stable industrial grade overall seawater splitting. *Chem. Eng. J.* **2024,** *488*, 150624.

11. Saquib, M.; Arora, P.; Bhosale, A., Nickel molybdenum selenide on carbon cloth as an efficient bifunctional electrocatalyst for alkaline seawater splitting. *Fuel* **2024,** *365*, 131251.

12. Gopalakrishnan, S.; Anandha Babu, G.; Harish, S.; Kumar, E. S.; Navaneethan, M., Interface engineering of heterogeneous NiMn layered double hydroxide/vertically aligned $NiCo_2S_4$ nanosheet as highly efficient hybrid electrocatalyst for overall seawater splitting. *Chemosphere* **2024,** *350*, 141016.

13. Su, X.; Shao, X.; Wang, Y.; Fan, W.; Song, C.; Wang, D., $CoNi_2S_4$ Nanosheets on Carbon Cloth Using a Deep Eutectic Solvent Strategy as Bifunctional Catalysts for Water/Simulated Seawater Electrolysis. *ACS Appl. Nano Mater.* **2023,** *6* (24), 23029-23036.

14. Liang, R.; Fan, J.; Lei, F.; Li, P.; Fu, C.; Lu, Z.; Hao, W., Fabrication of ultra-stable and high-efficient CoP-based electrode toward seawater splitting at industrial-grade current density. *J. Colloid Interface Sci.* **2023,** *645*, 227-240.
15. Zhang, X.; Shi, X. R.; Wang, P.; Bao, Z.; Huang, M.; Xu, Y.; Xu, S., Bio-inspired design of NiFeP nanoparticles embedded in (N,P) co-doped carbon for boosting overall water splitting. *Dalton. Trans.* **2023,** *52* (20), 6860-6869.
16. Yang, P.; Zhang, Z.; Jin, C.; Ren, M.; Wang, J.; Shi, T.; Xing, H.; Ji, X., Synthesis of Urchin-like Ni@NP@NCP Composites with Three Solvothermal Systems for Highly Efficient Overall Seawater Splitting. *Langmuir* **2023,** *39* (17), 6240-6248.
17. Hoa, V.; Austeria, M.; Thi Dao, H.; Mai, M.; Kim, D., Dual-phase cobalt phosphide/phosphate hybrid interactions via iridium nanocluster interfacial engineering toward efficient overall seawater splitting. *Appl. Catal. B Environ.* **2023,** *327*, 122467.
18. Sun, J.; Li, J.; Li, Z.; Li, C.; Ren, G.; Zhang, Z.; Meng, X., Modulating the Electronic Structure on Cobalt Sites by Compatible Heterojunction Fabrication for Greatly Improved Overall Water/Seawater Electrolysis. *ACS Sustain. Chem. Eng.* **2022,** *10* (30), 9980-9990.
19. Yang, P.; Ren, M.; Jin, C.; Xing, H., Facile Synthesis of N and P Co-Doped $NiMoO_4$ Hollow Nanowires and Electrochemical Deposition of NiFe-Layered Double Hydroxide for Boosting Overall Seawater Splitting. *J. Electrochem. Soc.* **2022,** *169* (4), 046511.
20. Wang, H.; Chen, L.; Tan, L.; Liu, X.; Wen, Y.; Hou, W.; Zhan, T., Electrodeposition of NiFe-layered double hydroxide layer on sulfur-modified nickel molybdate nanorods for highly efficient seawater splitting. *J. Colloid Interface Sci.* **2022,** *613*, 349-358.
21. Chen, W.; Wei, W.; Li, F.; Wang, Y.; Liu, M.; Dong, S.; Cui, J.; Zhang, Y.; Wang, R.; Ostrikov, K.; Zang, S., Tunable Built-In Electric Field in Ru Nanoclusters-Based Electrocatalyst Boosts Water Splitting and Simulated Seawater Electrolysis. *Adv. Funct. Mater.* **2023,** *34* (7), 2310690.
